# Bone Benefits After Simultaneous Pancreas-kidney Transplantation Compared With the Pretransplant Period

**DOI:** 10.1097/TP.0000000000005627

**Published:** 2026-02-09

**Authors:** Simona Kratochvílová, Jana Brunová, Petr Wohl, Michal Kahle, Peter Girman, František Saudek

**Affiliations:** 1 Diabetes Centre, Institute for Clinical and Experimental Medicine, Prague, Czech Republic.; 2 Department of Data Analysis, Statistics, and Artificial Intelligence, Institute for Clinical and Experimental Medicine, Prague, Czech Republic.

## Abstract

**Background.:**

Bone health is frequently compromised in patients with type 1 diabetes and advanced diabetic kidney disease. While simultaneous pancreas-kidney transplantation (SPKT) is the treatment of choice for selected patients, concerns remain about its skeletal impact, particularly because of immunosuppressive regimens.

**Methods.:**

We conducted a retrospective intraindividual comparison of bone mineral density (BMD) and trabecular bone score (TBS) before and after SPKT in 48 patients (mean age 41.5 ± 10.1 y) managed under a corticosteroid-sparing immunosuppressive protocol (tacrolimus + mycophenolate mofetil/sirolimus + prednisone only 4 wk after SPKT). Dual-energy X-ray absorptiometry scans were assessed at 3 time points: before listing, peritransplant (within 28 d from the date of SPKT, both before and after), and 2 y posttransplant. Annualized changes in BMD and TBS were analyzed along with predictors of bone outcomes.

**Results.:**

During the pretransplant period, BMD declined significantly at the femoral neck (–0.011 g/cm^2^/year; 95% confidence interval [CI],–0.019 to –0.003) and TBS decreased by –0.032/year (95% CI, –0.049 to –0.014). After SPKT, lumbar spine BMD increased (+0.039 g/cm^2^/year; 95% CI, 0.028-0.050), TBS improved (+0.019/year; 95% CI, –0.000 to 0.039), and femoral neck BMD stabilized. Distal radius BMD declined posttransplant (–0.011 g/cm^2^/year; 95% CI, –0.018 to –0.004). Trend differences between pretransplant and posttransplant periods were significant for lumbar spine BMD (*P* < 0.001), femoral neck BMD (*P* = 0.01), and TBS (*P* = 0.003).

**Conclusions.:**

SPKT under a corticosteroid-sparing regimen not only halts but may reverse bone loss at trabecular rich sites in type 1 diabetes with advanced diabetic kidney disease. In particular, the increase in BMD in the lumbar spine can be considered clinically significant. Our data support the strategy of early referral for SPKT in eligible patients.

## Graphical abstract

**Figure:**
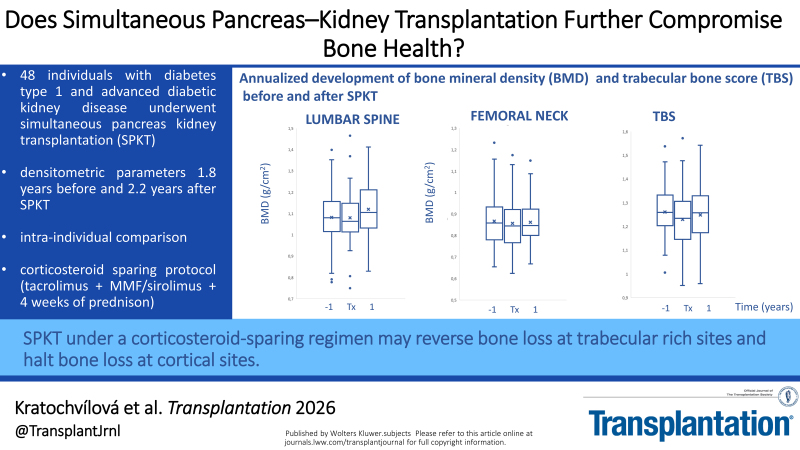

## INTRODUCTION

Type 1 diabetes (T1DM) and chronic kidney disease (CKD) are both important risk factors for bone metabolism impairment. Prevalence of osteoporosis (OP) is very high in individuals with T1DM and terminal stages of diabetic kidney disease (DKD) according to densitometric examination (dual-energy X-ray absorptiometry [DXA]), as demonstrated in our previous study of patients on the waiting list (WL) for simultaneous pancreas-kidney transplantation (SPKT).^1^ Only 15.8% of participants had normal bone mineral density (BMD) across all examined sites. On contrary 27.7% of participants had BMD within osteoporotic range at least in 1 region. The prevalence of OP increased significantly with the progression of DKD. However, the literature lacks an analysis of BMD development in T1DM patients in the terminal stages of DKD. Additionally, predictive factors have yet to be established.

SPKT is the treatment of choice for selected patients with T1DM and end-stage DKD.^2^ During the early posttransplant period, intensive induction immunosuppressive therapy, including high-dose corticosteroids, may exacerbate bone deterioration.^3^ Other factors contributing to the continued decline in BMD include the potential suboptimal functioning of both grafts, persistent vitamin D insufficiency, and parathyroid disease.^4-6^ The early posttransplant period has therefore traditionally been perceived as a high-risk period for bone metabolism. The results of studies conducted in individuals after SPKT are not consistent; recent reports highlight increases in BMD, challenging the conventional wisdom.^7-12^ At the same time, it must be taken into account that current progress in transplant therapy is so significant (new surgical techniques, changes in immunosuppressive protocols, comprehensive posttransplant care) that the results of some older studies will not be fully applicable to the current population.

Bone mineral quantity and bone microarchitecture quality are both crucial elements in fracture risk. New markers, such as the trabecular bone score (TBS), have recently been introduced as indirect indicators of bone microarchitecture, offering predictive value for fragility fractures independent of BMD.^13^ Previous reports have highlighted a reduction in TBS in patients with both T1DM^14,15^ and CKD as well as in kidney transplant recipients.^16-18^ To our knowledge, no data on the development of TBS in T1DM patients with DKD have been published, and studies on the development of TBS after kidney transplantation deliver conflicting outcomes.^10,19-21^

With regard to BMD loss, the hip is the most affected site in patients with CKD as well as in those with T1DM.^22^ Unsurprisingly, fracture of the hip results in the most devastating consequences. DXA-derived advanced hip analysis (AHA) facilitates assessment of hip geometry (cortical thickness of the femoral neck [FN], shaft, and calcar) and strength (strength index and buckling ratio). Decreases in these parameters have already been reported in the general CKD population, with a more pronounced deterioration demonstrated in T1DM patients.^23^ Additionally, the fracture rate has previously been correlated with the buckling ratio and cortical FN thickness.^24^

Given that SPKT is the treatment of choice for all eligible patients and should be performed at the earliest possible stage, performing a direct randomized comparison of patients with end-stage DKD and those post-SPKT was not feasible. Similarly, T1DM patients not indicated for SPKT because of medical reasons or advanced age are an unsuitable control group for comparison purposes. Given these limitations, we opted for a retrospective design comparing the above parameters before and after SPKT in the same group.

The aims of our study were:

1. to characterize the progression of BMD, TBS, and AHA parameters in patients with T1DM and end-stage DKD and to identify predictors of their deterioration. We hypothesized that both BMD and TBS would exhibit a markedly accelerated decline in this population.
2. to directly compare changes in these parameters during the pretransplant and posttransplant periods, and to test the hypothesis that SPKT with corticosteroid-sparing immunosuppressive protocol (tacrolimus + mycophenolate mofetil/sirolimus + prednisone only 4 wk after SPKT) confers a net skeletal benefit in spite of the potential risk factors associated with transplantation.

## MATERIALS AND METHODS

### Study Design and Patients

We reassessed a cohort of SPKT candidates first placed on the transplant WL between 2011 and 2016, as detailed in our previous study.^1^ Eligibility criteria for WL inclusion were age under 65, CKD stage G4–5 or 5D, and the absence of advanced cardiovascular disease, malignancy, active infection, or other absolute contraindications.

**FIGURE 1.**
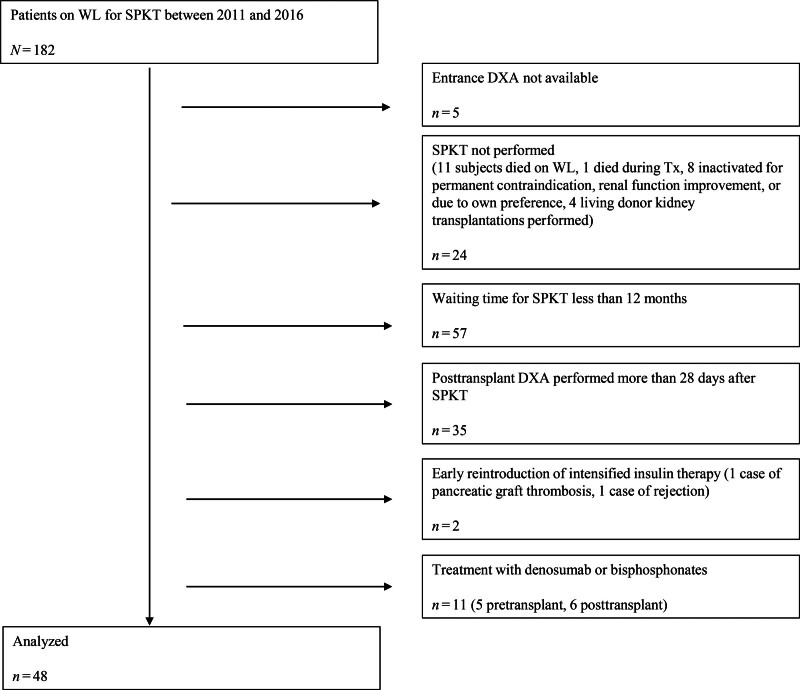
Patient selection flow chart. DXA, dual-energy X-ray absorptiometry; SPKT, simultaneous pancreas-kidney transplantation; Tx, transplant; WL, waiting list.

In the current analysis, we included 48 patients who fulfilled the following criteria: (1) on the WL for 12 mo or longer, (2) available DXA scan within ±28 d of the date for SPKT, both before and after (3) follow-up DXA performed approximately 2 y post-SPKT, and (4) no antiresorptive treatment, such as bisphosphonates or denosumab, before or after transplantation. All but 1 patient had T1DM; 1 individual had undergone subtotal pancreatectomy because of acute pancreatitis. A flow diagram illustrating the patient selection protocol is shown in Figure 1. All but 4 patients remained insulin-independent at follow-up. Two individuals with early pancreatic graft failure—because of thrombosis at 7 d and rejection at 1.5 mo—were excluded. However, we included 1 subject who underwent pancreatic graft explantation because of thrombosis at 22 mo and another who resumed basal insulin after rejection at 28 mo post-SPKT because the response of bone tissue to metabolic changes is slow and they spent the majority of the study period with functioning pancreatic graft.

Collected data included epidemiological characteristics (age, sex, age at diabetes onset, diabetes duration, dialysis vintage), anthropometric parameters, medical history, pharmacologic treatment, biochemical profiles, and densitometric measures.

The median interval between baseline and peritransplant DXA was 658 d (range, 386–1439 d), and the interval between peritransplant and follow-up DXA was 786.5 d (range, 420–1026 d). For statistical purposes, annualized BMD changes were calculated as follows: ΔBMD/days × 365; these changes were expressed as percentages.

The study was approved by the Ethics Committee of the Institute for Clinical and Experimental Medicine and Thomayer Hospital, Prague (approval no. A-19-05, issued February 13, 2019). Informed consent was waived because of the retrospective nature of the study and full anonymization of patient data.

### Transplantation Procedure

SPKT procedures were performed between August 2012 and January 2018 using deceased donor grafts with portal venous and enteric exocrine drainage. A standard immunosuppressive regimen was applied.^25^ Induction therapy consisted of 2 doses of methylprednisolone (500 and 250 mg) and 3–4 doses of anti-T-lymphocyte globulin. Maintenance immunosuppression included tacrolimus combined with either mycophenolate mofetil or sirolimus. Oral prednisone (initially 20 mg daily) was tapered and discontinued within 4 wk unless reinstituted because of rejection episodes or intolerance to other agents. Acute rejections were treated with methylprednisolone pulses or polyclonal anti-T-cell antibodies.

### Laboratory Measurements

Total serum calcium, phosphate, creatinine, and alkaline phosphatase (ALP) were measured spectrophotometrically using automated analyzers. Intact parathyroid hormone (PTH) was measured using an electrochemiluminescence immunoassay (Elecsys PTH, Roche Diagnostics, Mannheim, Germany). Serum 25-hydroxyvitamin D was measured by radioimmunoassay (DIAsource ImmunoAssays, Louvain-la-Neuve, Belgium), and glycosylated hemoglobin by high-performance liquid chromatography standardized to the reference method recommended by the International Federation of Clinical Chemistry and Laboratory Medicine^26^; values aligning with both the International Federation of Clinical Chemistry and Laboratory Medicine and the Diabetes Control and Complications Trial are reported. The estimated glomerular filtration rate was calculated using the 2009 Chronic Kidney Disease Epidemiology Collaboration formula.^27^

### Bone Densitometry

All participants underwent DXA of the lumbar spine (LS; L1–L4), total hip (TH), and FN and in the 33% distal radius (DR). The same DXA scanner (Lunar Prodigy Primo; GE Healthcare, Madison, WI) was used throughout the study. Daily instrument calibration was performed using a standard spine phantom. Measurements were analyzed using enCORE software version 13.60.033 (GE Healthcare), referencing the combined NHANES/Lunar database for the U.S. population.

Results were expressed as absolute values (g/cm^2^) and Z scores (based on age- and sex-specific healthy population norms), as the majority of study participants was younger than 50 y old. A Z score ≤ –2.0 was classified as below the expected range for age. DR BMD results were missing in 5 patients pretransplant and in 2 patients peritransplant. TBS was derived from LS scans using TBS iNsight software version 3.0.3.0 (GE Healthcare). TBS values were categorized as normal (>1.31) or high-risk (<1.23) according to McCloskey et al.^28^

Structural AHA included measurements of FN width, cortical widths of the neck, shaft, and calcar, strength index, and the buckling ratio using enCORE software version 18. The hip strength index was calculated based on a model developed by Yoshikawa et al.^29^ Buckling ratio was calculated as FN radius divided by its cortical thickness. Mean values from both sides were used for analysis.

### Statistical Analysis

Data are presented as the mean ± SD or median (range). All confidence intervals (CIs) are 95%. Paired *t* tests or Wilcoxon signed-rank tests were used for comparisons depending on normality (assessed using Q-Q plots). Linear regression models were fitted using ordinary least squares. No multiple testing correction was applied because the study was exploratory and focused on hypothesis generation. However, the risk of inflated type I errors because of multiple comparisons should be acknowledged. All analyses were performed using Python and StatsModels packages.

## RESULTS

Men predominated in our study cohort (n = 35; 73%). Mean age at the beginning of the pretransplant evaluation was 41.5 ± 10.1 y. The average age at diabetes onset was 15.4 ± 9.5 y, with a mean diabetes duration of 26.2 ± 8.5 y. At baseline, only 18 participants were on dialysis; this number increased to 37 at the time of transplantation, categorized as follows: hemodialysis in 24, peritoneal dialysis in 9, and both modalities sequentially in 4. Among those on dialysis, the median dialysis vintage was 20 mo.

Mean body mass index (BMI) decreased from 25.1 ± 3.9 kg/m^2^ at study entry to 24.2 ± 3.3 kg/m^2^ at the time of transplantation (*P* = 0.008), before increasing post-SPKT to 25.5 ± 4.1 kg/m^2^ (*P* = 0.001). Laboratory parameters at baseline, peritransplant, and posttransplant time points are summarized in Table 1. Bone active pharmacotherapy is listed in Table 2. One subject underwent subtotal parathyroidectomy before the pretransplant workup.

**TABLE 1.** Pretransplant, peritransplant, and posttransplant laboratory parameters

Parameter	Pretransplant^*a*^	Peritransplant^*b*^	Posttransplant^*c*^
Calcium (mmol/L)	2.24 ± 0.18	2.4 ± 0.2	2.5 ± 0,1
Phosphorus (mmol/L)	1.64 ± 0.39	1.13 ± 0.6 (n = 46)	1.0 ± 0.2
Creatinine (µmol/L)	437.4 ± 227.4	609.6 ± 270.2	131.5 ± 43.8
CK-EPI (mL/s/1.73 m^2^)	0.28 ± 0.17	0.17 ± 0.08	0.96 ± 0.31
HbA1c IFCC (mmol/mol)	75.4 ± 18.6	71.2 ± 15.5	37.8 ± 6.4
DCCT (%)	9.1 ± 1.7	8.7 ± 1.4	5.6 ± 0.6
Glycemia (mmol/L)	N/A	N/A	5.4 ± 0.9
PTH (pmol/L)	18.3 ± 11.9 (n = 46)	20.4 ± 12.4 (n = 46)	12.7 ± 9.2 (n = 47)
Vitamin D (µg/L)	17.0 ± 8.5 (n = 39)	13.3 ± 5.5 (n = 44)	26.9 ± 10.5 (n = 35)
ALP (µkat/l)	1.5 ± 0.5	2.4 ± 0.2	1.7 ± 0.7

a Pretransplant investigation, on average 1.8 y before SPKT.

b Calcium, creatinine, CK-EPI, HbA1c, and ALP levels were assessed on the day of transplantation; phosphorus, PTH, and vitamin D levels were assessed on day 8 posttransplant.

c Posttransplant annual checkup, on average 2.2 y after SPKT.

ALP, alkaline phosphatase; CKD-EPI, Chronic Kidney Disease Epidemiology Collaboration; DCCT, Diabetes Control and Complications Trial; HbA1c, glycosylated hemoglobin; IFCC, International Federation of Clinical Chemistry and Laboratory Medicine; N/A, not applicable; PTH, intact parathyroid hormone; SPKT, simultaneous pancreas-kidney transplantation.

**TABLE 2.** Number of patients given bone-modulating therapy pretransplant, peritransplant, and posttransplant

Treatment	Pretransplant^*a*^	Peritransplant^*b*^	Posttransplant^*c*^
Calcium^*d*^	20	14	26
Phosphate binders	7	27	0
Any form of vitamin D	24	37	33
Calcitriol	19	26	3
Paricalcitol	4	8	0
Cholecalciferol	5	12	31
Calcimimetics	0	7	3
Treatment-naive	11	1	10

a Pretransplant investigation, on average 1.8 y before SPKT.

b Peritransplant, upon admission for SPKT.

c Posttransplant, on average 2.2 y after SPKT.

d Calcium administration included calcium binders and thiazide diuretics.

SPKT, simultaneous pancreas-kidney transplantation.

All participants received tacrolimus-based immunosuppression, combined with mycophenolate mofetil in 28 cases and sirolimus in 19 cases; 1 patient received both mycophenolate mofetil and sirolimus sequentially. Prolonged prednisone therapy (cumulative exposure >6 mo) was required in 10 individuals, in most cases because of intolerance to maintenance immunosuppression (neutropenia). For rejection episodes, methylprednisolone pulses were administered in 12 patients.

**FIGURE 2.**
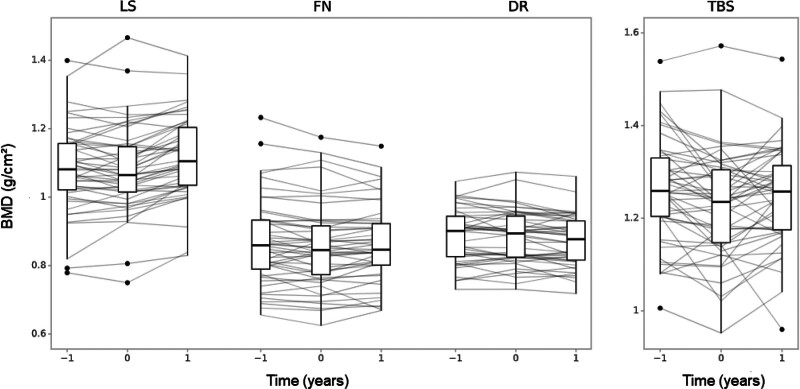
Comparison of the annualized development of selected parameters pretransplant and posttransplant. Boxplots for BMD of the LS, FN, and DR as well as for the TBS are shown at the time of transplantation, before and after. BMD, bone mineral density; DR, distal radius; FN, femoral neck; LS, lumbar spine; TBS, trabecular bone score.

Changes in BMD and AHA parameters during the pretransplant and posttransplant periods are shown in Table 3. LS BMD remained stable pretransplant but increased post-SPKT by 0.039 g/cm^2^/year (95% CI, 0.028-0.050). FN BMD declined during the waiting period by –0.011 g/cm^2^/year (95% CI, –0.019 to –0.003) but stabilized after transplantation. DR BMD declined only in the posttransplant period by –0.011 g/cm^2^/year (95% CI, –0.018 to –0.004). TBS decreased pretransplant by –0.032/year (95% CI, –0.049 to –0.014), followed by a posttransplant recovery of +0.019/year (95% CI, –0.000 to 0.039). No significant changes in AHA parameters were observed in either period. Annualized BMD percentage changes are given in Table 4. Differences in BMD trends between periods, which were statistically significant for LS (*P* < 0.001), FN (*P* = 0.01), and TBS (*P* = 0.003), are illustrated in Figure 2.

**TABLE 3.** Pretransplant, peritransplant, and posttransplant absolute values and annualized development for parameters of bone mineral density and advanced hip analysis

Parameter	Pretransplant value^*a*^	Peritransplant value^*b*^	Posttransplant value^*c*^	Pretransplant development	Posttransplant development
Lumbar spine (g/cm^2^)	1.092 ± 0.134	1.081 ± 0.127	1.163 ± 0.121	–0.002 (–0.014 to 0.010)	0.039 (0.028-0.050)
(Z score)	–0.85 ± 1.19	–0.95 ± 1.14	–0.21 ± 1.06		
Total hip (g/cm^2^)	0.917 ± 0.137	0.894 ± 0.135	0.901 ± 0.118	–0.007 (–0.015 to 0.001)	0.003 (–0.005 to 0.011)
(Z score)	–0.89 ± 1.06	–1.04 ± 1.03	–0.94 ± 0.89		
Femoral neck (g/cm^2^)	0.883 ± 0.140	0.857 ± 0.123	0.871 ± 0.109	–0.011 (–0.019 to –0.003)	0.005 (–0.003 to 0.013)
(Z score)	–0.91 ± 1.14	–1.08 ± 1.02	–0.89 ± 0.95		
Distal radius (g/cm^2^)	0.893 ± 0.086	0.884 ± 0.078	0.863 ± 0.077	–0.002 (–0.010 to 0.006)	–0.011 (–0.018 to –0.004)
(Z score)	–0.75 ± 0.85	–0.81 ± 0.77	–1.0 ± 0.80		
Trabecular bone score	1.292 ± 0.120	1.229 ± 0.114	1.271 ± 0.117	–0.032 (–0.049 to –0.014)	0.019 (–0.000-0.039)
Cortical thickness—neck (mm)	6.22 ± 2.08	6.01 ± 2.94	5.86 ± 1.63	0.076 (–0.241 to 0.394)	–0.054 (–0.363 to 0.254)
Shaft	5.20 ± 1.59	5.18 ± 1.99	4.59 ± 1.73	–0.047 (–0.359 to 0.264)	–0.351 (–0.713 to 0.011)
Calcar	3.77 ± 1,38	4.00 ± 1.28	3.94 ± 1.21	0.146 (–0.133 to 0.425)	0.009 (–0.200 to 0.217)
Femoral neck width	33.4 ± 3.47	33.65 ± 3.92	33.44 ± 3.37	0.172 (–0.073 to 0.417)	–0.114 (–0.273 to 0.046)
Strength index	1.48 ± 0.43	1.52 ± 0.39	1.50 ± 0.45	0.029 (–0.026 to 0.084)	–0.015 (–0.067 to 0.037)
Buckling ratio	3.48 ± 1.38	3.78 ± 1.6	3.71 ± 1.34	0.087 (–0.137 to 0.311)	–0.036 (–0.244 to 0.171)

Pretransplant, peritransplant, and posttransplant values are listed as the mean ± SD; pretransplant and posttransplant annualized development are listed as the mean with 95% confidence interval.

a Pretransplant investigation, on average 1.8 y before SPKT.

b Peritransplant examination, on average 13 d after SPKT.

c Posttransplant annual checkup, on average 2.2 y after SPKT.

SPKT, simultaneous pancreas-kidney transplantation.

**TABLE 4.** Comparison of pretransplant and posttransplant development of bone mineral density parameters

Parameter	Pretransplant period	Posttransplant period	*P*
Lumbar spine (%)	0.03 ± 3.95	3.79 ± 3.73	<0.001
Total hip (%)	–0.67 ± 2.96	0.58 ± 3.03	0.110
Femoral neck (%)	–1.12 ± 3.16	0.78 ± 3.35	0.010
Distal radius (%)	–0.11 ± 2.88	–1.26 ± 2.63	0.092
Trabecular bone score (%)	–2.24 ± 4.52	1.79 ± 5.72	0.003

Values are annualized and expressed as the mean ± SD.

The number of patients with a Z score ≤ –2.0 at 1 or more of the major osteoporotic sites (LS, TH, or FN) declined from 14 pretransplant to 12 peritransplant and to 8 posttransplant. The number of participants with high-risk TBS (<1.23) increased from 13 pretransplant to 23 peritransplant and subsequently declined to 18 posttransplant. According to T scores, OP (T score ≤ –2.5) at least at 1 major osteoporotic site (LS, TH, or FN) during the pretransplant examination was present in 10 participants (specifically in 4 in LS, 3 TH, and 8 FN), persisted in 9 participants to the date of SPKT (3, LS, 5 TH, 7 FN) and decreased to 7 individuals 2 y after (2 LP, 4 TH, 7 FN).

We evaluated the impact of selected clinical and laboratory variables on changes in LS, TH, FN, and DR BMD as well as TBS using linear regression models. Variables included age, sex, age at diabetes onset, total dialysis vintage, proportion of time on dialysis, BMI (absolute and delta), bone medications (calcium including thiazides, cholecalciferol, any form of vitamin D, calcimimetics, phosphate binders), corticosteroid exposure (prednisone >6 mo, methylprednisolone pulses), glycosylated hemoglobin, estimated glomerular filtration rate, immunosuppressive regimen (mycophenolate mofetil versus sirolimus), PTH, ALP, and 25-hydroxyvitamin D.

Longer dialysis vintage was associated with a decline in pretransplant LS BMD (–0.0023 g/cm^2^/month; 95% CI, –0.005 to –0.000047; *P* = 0.046). Prolonged corticosteroid therapy was associated with posttransplant TH BMD loss (–0.0235 g/cm^2^; 95% CI,–0.044 to –0.003; *P* = 0.028), as were methylprednisolone pulses (–0.0230 g/cm^2^; 95% CI,–0.041 to –0.005; *P* = 0.014). Conversely, an increase in BMI posttransplant was positively associated with TH BMD gain (+0.0038 g/cm^2^ per 1 kg/m^2^; 95% CI, 0.000-0.008; *P* = 0.041). Immunosuppression type (mycophenolate versus sirolimus) did not have any significant effect on bone measures’ development after the transplantation.

Finally, 4 patients experienced fractures during the posttransplant follow-up (DR, patella, toe, and metatarsal), on average 11 mo posttransplant. All fractures were traumatic and occurred after a fall from a standing position. Two individuals had both OP and high-risk TBS at the time of transplantation, other 2 had osteopenia, in 1 case with normal TBS and in another case with high-risk TBS. However, the sample size was too small to allow for meaningful statistical analysis.

## DISCUSSION

We present a unique intraindividual comparison of the evolution of BMD, TBS, and AHA parameters during the terminal stages of DKD and after SPKT in the same cohort of patients with T1DM. The burden of skeletal pathology was substantial at baseline, with 14 patients (29%) presenting with pathological Z scores and 13 patients (27%) with TBS values in the high-risk range. The pretransplant decline in FN BMD and TBS was followed by a posttransplant increase in LS BMD and TBS. The most striking difference was observed in the pretransplant and posttransplant trajectories of the rapidly remodeling trabecular bone at the LS.

### Pretransplant Period

Bone disease in SPKT candidates during the pretransplant period is of multifactorial pathogenesis. T1DM negatively affects both BMD and bone quality.^30^ The pathophysiology of bone impairment in T1DM includes several mechanisms: toxic effect of chronic hyperglycemia, reduced anabolic effects of insulin and insulin-like growth factor 1 (both critical for achieving peak bone mass), accumulation of advanced glycation end-products in the bone matrix, autonomic neuropathy, autoimmune inflammation, and increased urinary calcium excretion.^22,30-32^

However, the presence of T1DM alone does not appear to account for the extensive bone loss observed in patients with advanced DKD. For example, studies conducted in individuals with T1DM and preserved renal function report little or no reduction in bone mass.^33-35^ Notably, impaired insulin clearance in patients with renal failure may result in improved glycemic control,^36^ which could potentially slow the progression of bone deterioration.

In contrast, progressive renal failure is associated with the development of Chronic Kidney Disease-Mineral and Bone Disorder (CKD-MBD), a complex disorder involving abnormalities in calcium, phosphate, PTH, vitamin D, and fibroblast growth factor 23 metabolism. These disturbances play a central role in the ensuing profound alterations in bone turnover, mineralization, and volume.^37^ Patients with CKD experience more rapid bone loss than the general population.^38^ Various reports describe a nonsignificant decrease in LS BMD but up to a 4.5% mean annual decline in TH and FN BMD in unselected older patients across different CKD stages.^39-42^

To our knowledge, the rate of bone loss in patients with DKD has only been studied in older individuals (mean age 65 y) at CKD stage G3 or G4, most of whom exhibited type 2 diabetes. In this population, T scores declined by 0.5 and 0.19 SDs at the total body and FN, respectively, over a 2-y follow-up period.^43^ The rate of bone loss observed in our high-risk cohort of patients with both T1DM and advanced complications did not exceed those reported in the literature.

Advances in pharmacologic management of CKD-MBD—targeting phosphate control, vitamin D supplementation, and PTH modulation—may help prevent further deterioration of mineral metabolism and, secondarily, improve bone outcomes. In our cohort, the proportion of treated individuals increased significantly from baseline to the time of transplantation (Table 2). However, these medications likely improve the bone mineralization rate rather than reverse microarchitectural damage. Of note, the TBS decline observed in our study exceeded the annual loss reported in a cohort of older, unselected dialysis patients (2.4% versus 1.95% annual decrease).^40^

### Posttransplant Period

Successful renal transplantation restores kidney function and initiates the gradual resolution of CKD-MBD-related abnormalities. However, full recovery of all components of mineral metabolism takes time, and elevated PTH levels often persist despite normalization of kidney function. A novel posttransplant feature—prolonged hypophosphatemia because of persistent elevation of fibroblast growth factor 23 further contributes to bone demineralization.^44^

A functioning pancreatic graft restores glucose homeostasis. This leads to a reduction in the acute detrimental effects of hyperglycemia on osteoblasts, interrupting advanced glycation end-products accumulation in the bone matrix and halting excessive urinary calcium loss. Emerging animal data suggest that C-peptide replacement therapy in streptozotocin-induced diabetic rats improves trabecular bone quality,^45^ indicating the potential direct role of C-peptide in bone metabolism. In addition, the overall metabolic improvement promotes anabolism. Dietary liberalization after transplantation frequently leads to increased BMI, and mechanical loading associated with weight gain has been shown to improve BMD in patients with type 2 diabetes.^46^ A similar mechanical effect might be expected in our cohort.

On the other hand, immunosuppressive therapy may exert negative effects on bone. Corticosteroids rapidly impair bone remodeling and reduce intestinal calcium absorption. Other immunosuppressive agents, including calcineurin inhibitors, sirolimus, and mycophenolate mofetil, do not appear to have clinically relevant adverse effects on bone.^44^

In our study, the most pronounced change following SPKT was to LS BMD, exhibiting a mean annual increase of 3.8%. The lumbar vertebrae largely consist of metabolically active trabecular bone, which is capable of rapidly responding to shifts in metabolic conditions. In general, patients after kidney transplantation may experience early bone loss of up to 10% at trabecular sites within the first 3 to 6 mo, followed by BMD stabilization beyond the first posttransplant year.^47^ One study that followed patients after SPKT (performed between 1995 and 1997) reported a rapid LS BMD decline of 6% >6 mo,^11^ while a more recent study observed a 0.4 T score (SD) decrease in LS BMD during the first year posttransplant.^9^ In contrast, 3 studies evaluating SPKT recipients transplanted after the year 2000 reported increases in LS BMD from the first posttransplant year onward.^7,8,10^

TBS increased by 1.8% per year after SPKT in our cohort in contrast to individuals after kidney transplant alone, where continuous TBS decline^19,20^ or no change^21^ during the first year posttransplant has been reported. Both the magnitude of LS BMD increase and TBS improvement suggest that patients receiving both pancreatic and kidney grafts experience more robust metabolic recovery compared with those after kidney transplant alone.

TH and FN, composed primarily of cortical bone, exhibit slower metabolic turnover and may be influenced by persistent secondary hyperparathyroidism. In our study, BMD declined gradually in both regions before transplantation and increased slightly afterward, although the difference reached statistical significance only for FN. In the 1990s, Smets et al^11^ reported a 4.3% FN BMD loss in the first 6 mo post-SPKT. Similar cortical bone losses of up to 8% were observed after kidney transplantation alone during that period.^47^ More recent SPKT studies (post-1998) describe either BMD stability or small delayed increases at the FN.^7-9^ In our previous study, TH and FN BMD remained unchanged during the first posttransplant year and began to rise thereafter.^10^

The DR was the only site that exhibited continuous BMD decline after SPKT. This phenomenon, which we previously documented,^10^ may reflect the combination of persistent hyperparathyroidism and limited mechanical loading at nonweight-bearing cortical bone sites in contrast to weight-bearing regions where improved mobility post-SPKT may drive BMD gain. However, the question of whether a decrease in DR BMD also translates into an increased risk at weight-bearing sites after SPKT still requires clarification.

In the current study, all AHA parameters remained stable during both the pretransplant and posttransplant periods, consistent with the inherently slower turnover of cortical bone.

The more favorable bone recovery observed in our study, unlike in some previous reports, may reflect current practices that prioritize lower cumulative corticosteroid exposure and a shift toward modern immunosuppressive agents. In our cohort, prolonged corticosteroid therapy or the need for corticosteroid pulses negatively predicted TH BMD development. Later studies^7-9,12^ also applied corticosteroid-sparing protocols but with delayed prednisone withdrawal in comparison with our praxis (4 wk after SPKT). All SPKT procedures were performed using enteric pancreatic drainage, avoiding the skeletal demineralization associated with metabolic acidosis induced by bladder drainage, a technique commonly used in the 1990s. It is worth noting that we observed an increase in LS BMD, even though, unlike some of the aforementioned studies,^8-10^ we excluded individuals treated with bisphosphonates and our study cohort was older than these in other studies (41.5 y at the beginning of pretransplant investigation versus 24–39 y at SPKT date).^7-9,11,12^

In addition to the above-mentioned reasons, modern posttransplant management emphasizes correction of vitamin D deficiency and preventive strategies for bone health. Specific recommendations for bone metabolism monitoring in SPKT candidates before and after transplantation are still lacking. Therefore, we have to rely on the general Kidney Disease: Improving Global Outcomes guidelines.^48^ In addition to these recommendations, we suggest that bone densitometry and assessment of all CKD-MBD components with addressing pathological values should become an integral part of the pretransplant workup. Future effective prehabilitation programmes should include counseling on appropriate diet, suitable physical activity and individualized fall prevention strategies.

In the early posttransplant period, a review of all measures taken should be carried out and bone densitometry should be updated if necessary. In the later posttransplant period, we recommend regular annual monitoring of calcium, phosphate, PTH, ALP, and vitamin D levels and repeat densitometry at 1- or 2-y intervals to identify individuals with low BMD or accelerated bone loss. Our data also suggest that calcium and vitamin D supplementation may be effective in cases of insufficient levels or low BMD. Larger studies are needed on the efficacy and safety of antiresorptive or osteoanabolic therapy after renal transplantation. In patients with low BMD, bone deteriorating medications should be avoided, and our data suggest that minimizing corticosteroid doses prevents further bone loss. Fractures after SPKT should be recorded and analyzed, and all preventive regime measures including muscle strengthening and weight-bearing exercise to enhance cortical sites recovery, should be implemented.

### Study Strengths and Limitations

Our study provides a unique intraindividual comparison of patients with T1DM in the terminal stage of DKD and after SPKT. Although the retrospective design of the study represents a limitation, it also reflects real-world clinical practice. We aimed to maximize cohort homogeneity and minimize confounding factors by excluding patients with pancreatic graft failure, those receiving antiresorptive therapy, and those exhibiting excessive variability in the timing of peritransplant DXA assessments (the median time from SPKT to DXA was 13 d). In addition, intraindividual design minimizes between-patient variability.

Observation duration for the pretransplant and posttransplant periods was not strictly equivalent, requiring the annualization of changes in bone parameters. This approach introduces a degree of simplification, as bone dynamics differ across CKD stages and vary between the first and second posttransplant years. Additionally, we cannot entirely exclude the influence of bone-modifying therapies administered during either period, although such treatment was generally reduced and streamlined after transplantation.

Only densitometry-derived parameters were available and, in the absence of bone biopsies, characterizing the specific type of bone pathology was not feasible. Moreover, physical activity levels – an important determinant of bone health—could not be reliably assessed retrospectively in individual patients. Similarly, fracture incidence was evaluated retrospectively and the lack of fracture adjudication (eg, in case of asymptomatic vertebral compression fractures) may underestimate total fracture incidence.

In conclusion, patients with T1DM in advanced stages of DKD frequently exhibit bone impairment involving both reduced BMD and compromised bone microarchitecture. In our study, the final 2 y preceding SPKT were not associated with accelerated bone loss, likely because of intensive monitoring and treatment of CKD-MBD components. The skeletal health of patients undergoing SPKT with a corticosteroid-sparing immunosuppressive protocol improved significantly. The most marked benefit occurred in metabolically active trabecular bone, despite persistent deficits in bone microarchitecture. Cortical bone loss halted at the FN and TH but continued at the DR.

## ACKNOWLEDGMENTS

The authors are grateful to Mr Michael FitzGerald for his help with language editing.
